# MyD88 in Macrophages Enhances Liver Fibrosis by Activation of NLRP3 Inflammasome in HSCs

**DOI:** 10.3390/ijms222212413

**Published:** 2021-11-17

**Authors:** Shuang Ge, Wei Yang, Haiqiang Chen, Qi Yuan, Shi Liu, Yongxiang Zhao, Jinhua Zhang

**Affiliations:** 1 National Center for International Research of Bio-Targeting Theranostics, Guangxi Key Laboratory of Bio-Targeting Theranostics, Collaborative Innovation Center for Targeting Tumor Diagnosis and Therapy, Guangxi Talent Highland of Bio-Targeting Theranostics, Guangxi Medical University, Nanning 530021, China; geshuang3322@163.com (S.G.); defzoe@163.com (W.Y.); 2 College of Life Science and Bioengineering, Beijing Jiaotong University, Beijing 100044, China; 20121605@bjtu.edu.cn (H.C.); 17118470@bjtu.edu.cn (Q.Y.); 18121615@bjtu.edu.cn (S.L.)

**Keywords:** MyD88, macrophage, HSC, liver fibrosis, NLRP3

## Abstract

Chronic liver disease mediated by the activation of hepatic stellate cells (HSCs) leads to liver fibrosis. The signal adaptor MyD88 of Toll-like receptor (TLR) signaling is involved during the progression of liver fibrosis. However, the specific role of MyD88 in myeloid cells in liver fibrosis has not been thoroughly investigated. In this study, we used a carbon tetrachloride (CCl_4_)-induced mouse fibrosis model in which MyD88 was selectively depleted in myeloid cells. MyD88 deficiency in myeloid cells attenuated liver fibrosis in mice and decreased inflammatory cell infiltration. Furthermore, deficiency of MyD88 in macrophages inhibits the secretion of CXC motif chemokine 2 (CXCL2), which restrains the activation of HSCs characterized by NLR Family Pyrin Domain Containing 3 (NLRP3) inflammasome activation. Moreover, targeting CXCL2 by CXCR2 inhibitors attenuated the activation of HSCs and reduced liver fibrosis. Thus, MyD88 may represent a potential candidate target for the prevention and treatment of liver fibrosis.

## 1. Introduction

Hepatic fibrosis results from sustained wound-healing responses to chronic liver injury in conjunction with the accumulation of extracellular matrix (ECM) [1]. The main causes of liver fibrosis include chronic hepatitis C virus (HCV) and hepatitis B virus (HBV) infection, alcoholic liver disease (ALD), and nonalcoholic steatohepatitis (NASH) [2,3,4]. If the injury is sustained, the excess deposition of ECM can alter nodule formation, interfere with hepatic function, and result in the end-stage consequence: cirrhosis [5,6]. It can lead to life-threatening complications of portal hypertension and liver failure and to the high risk of incident hepatocellular carcinoma (HCC) [7]. 

HSCs represent the major liver mesenchymal cell type playing a key role in fibrosis. HSCs reside in the space of Disse in the liver and store vitamin A lipids as retinyl ester [8,9]. Quiescent HSCs (qHSCs) are activated upon liver injury, with changes in gene expression and major phenotypical transformation to a-smooth muscle actin (α-SMA)-positive myofibroblasts that increase cell proliferation and migration, produce large amounts of collagen and other ECM molecules and secrete proinflammatory cytokine [10,11].

Immune cells, including myeloid cells, play critical functions in liver inflammation and fibrosis [2,12,13]. As widely known, macrophages are myeloid lineage cells that arise from bone marrow-derived monocytic progenitor cells. Macrophages are central players in liver fibrosis and exert bidirectional roles in the regulation of matrix deposition and resolution. It is clear that macrophages can play fibrogenic and fibrolytic roles due to their heterogeneity and plasticity [14,15]. Both monocyte-derived macrophages and Kupffer cells promote fibrogenesis by secreting transforming growth factor (TGF-β) and galectin-3, which drive transdifferentiation of HSCs into matrix-secreting myofibroblasts [16,17]. Different cell types in the liver make a network and relate to hepatic fibrogenesis regulation [18]. Bone marrow-derived macrophages (BMDMs) serve as the main cell subset producing pro-inflammatory cytokines [19].

TLRs are critical receptors and signal transducers for structurally conserved pathogen-associated molecular patterns (PAMPs) [20]. Except for TLR3, MyD88 is a dependent pathway for all TLRs to activate the NF-kB inflammation pathway [21]. Activation of MyD88 pathway has been reported in hepatic fibrotic diseases. MyD88 deficiency significantly reduces liver fibrosis and decreases eosinophil percentage in vivo [20,22]. Targeted deletion of B-cell-intrinsic MyD88 signaling resulted in reduced infiltration of migratory CD11c+ dendritic cells and Ly6C+ monocytes and hence reduced liver fibrosis [23]. In addition, inhibition of MyD88 led to the inhibition of HSC activation in vitro [24]. To date, the specific function of MyD88 in macrophages in liver fibrosis has not been reported. In this study, we found that MyD88 deficiency in myeloid cells attenuated CCl_4_-induced liver fibrosis. Furthermore, MyD88 in macrophages enhanced CXCL2 secretion and activated NLRP3 inflammasome in HSCs, which in turn promoted liver fibrosis.

## 2. Results

### 2.1. MyD88 Expression in Macrophages Is Upregulated during the Progression of Liver Fibrosis

**Figure 1:**
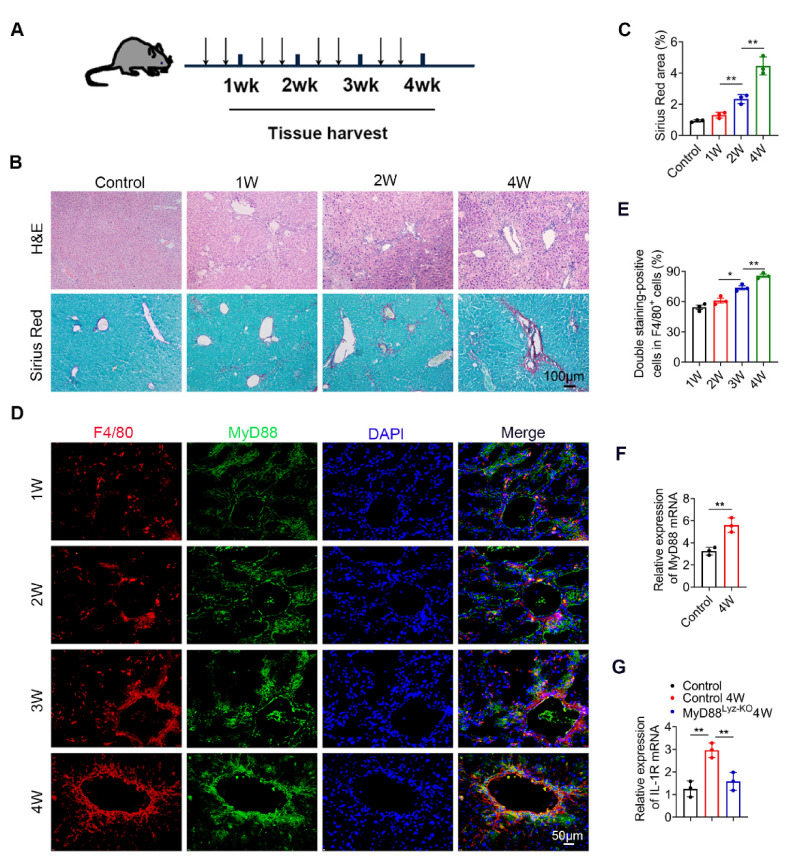
MyD88 expression in macrophages is upregulated during the progression of liver fibrosis. Groups of C57BL/6 mice (*n* = 5 per group) were subjected to CCl_4_-induced liver fibrosis. The data are representative of at least three independent experiments. (**A**) Schematic illustration of CCl_4_-induced liver fibrosis. (**B**) Liver tissues were harvested at the indicated time points and stained with H&E, Sirius Red. Scale bar, 100 μm. (**C**) Statistical analysis of Sirius Red staining in liver tissues. ** *p* < 0.01. (**D**,**E**) Representative double staining and statistical analysis of F4/80 and MyD88 in liver tissues. Scale bar, 50 μm. * *p* < 0.05 and ** *p* < 0.01. (**F**) Fold change of MyD88 mRNA in isolated BMDMs from control and 4W CCL_4_ treated mice. ** *p* < 0.01. (**G**) Fold change of IL-1R mRNA in isolated BMDMs. ** *p* < 0.01.

To investigate the role of MyD88 during liver fibrosis, C57BL/6 mice were administered with CCl_4_. Liver tissues were harvested at various time points in the progression of liver fibrosis, as shown in Figure 1A. The degree of liver fibrosis was enhanced and collagen deposition was significantly increased in livers during the progression of liver fibrosis, as indicated by H&E staining and Sirius Red staining (Figure 1B,C). Double immunofluorescence staining revealed that MyD88 was highly expressed in F4/80^+^macrophagesin fibrotic liver tissues (Figure 1D,E). Then we isolated BMDMs from control and 4W CCL_4_ treated mice, qPCR analysis revealed that MyD88 and IL-1R were also increased significantly after CCl_4_ treatment (Figure 1F,G). In addition, qPCR analysis showed that IL-1R was decreased significantly in MyD88^Lyz-KO^mice (Figure 1G). The obtained results suggest that MyD88 is significantly upregulated in macrophages during the progression of liver fibrosis. These results suggest that MyD88 in macrophages may play an important role in liver fibrosis.

### 2.2. MyD88 Deficiency in Myeloid Cells Attenuates Liver Fibrosis in Mice

**Figure 2:**
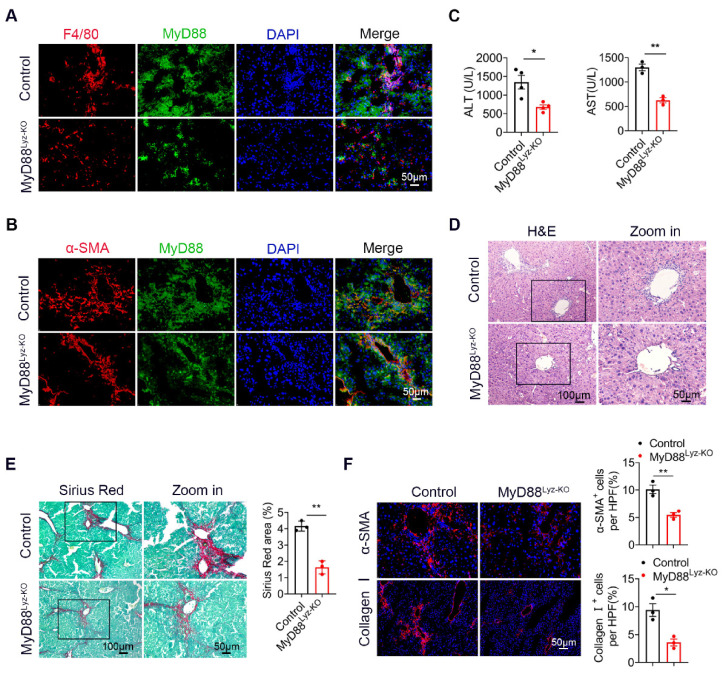
Myeloid cell-specific MyD88 deletion attenuates CCl_4_-induced liver fibrosis. (**A**,**B**) Representative immunofluorescence analysis of MyD88 expression in macrophages and myofibroblasts from CCl_4_-treated control and MyD88^Lyz-KO^ mice. F4/80 and α-SMA were used as cell-specific markers. Scale bar, 50 μm. (**C**–**F**) Groups of control and MyD88^Lyz-KO^ mice (*n* = 5 per group) were subjected to CCl_4_-induced liver fibrosis. The data are representative of at least three independent experiments. (**C**) Serum ALT and AST levels. * *p* < 0.05 and ** *p* < 0.01. (**D**) Representative staining of H&E in liver tissues. Scale bar, 100 μm (left). Scale bar, 50 μm (right). (**E**) Liver tissues were harvested and stained with Sirius Red. Scale bar, 100 μm (left). Scale bar, 50 μm (right). Statistical analysis of Sirius Red staining in liver tissues. ** *p* < 0.01. (**F**) Representative staining and statistical analysis of α-SMA and collagen I in liver tissues. Scale bar, 50 μm. * *p* < 0.05 and ** *p* < 0.01.

To evaluate the effect of MyD88 signaling in myeloid cells in liver fibrosis, we generated mice lacking MyD88 in myeloid cells (MyD88^Lyz-KO^ mice). Transgenic mice expressing Cre recombinase from the lyz promoter were crossed with MyD88^flox/flox^ mice. The lyz-Cre MyD88^flox/flox^ conditional knockout mice were designated MyD88^Lyz-KO^ mice, whereas their littermate single-transgenic mice were used as control mice. The absence of MyD88 in myeloid cells from the MyD88^Lyz-KO^ mice was demonstrated by double immunofluorescence staining (Figure 2A,B). MyD88^Lyz-KO^ mice and control mice were treated with CCl_4_ for 4 weeks. As shown in Figure 2C, the serum levels of ALT and AST were significantly decreased in CCl_4_-treated MyD88^Lyz-KO^ mice compared to the CCl_4_-treated control mice. Furthermore, CCl_4_-treated MyD88^Lyz-KO^ mice exhibited weakened liver injury and fibrosis compared with CCl_4_-treated control mice as assessed by H&E staining (Figure 2D) and Sirius Red staining (Figure 2E). Consistently, less hepatic expression of α-SMA and collagen I were found in CCl_4_-treated MyD88^Lyz-KO^ mice (Figure 2F). These results indicate that MyD88 deletion in myeloid cells attenuates liver injury and inhibits CCl_4_-induced liver fibrosis.

### 2.3. MyD88 Deficiency in Myeloid Cells Decreases Inflammatory Cell Infiltration in the Liver

**Figure 3:**
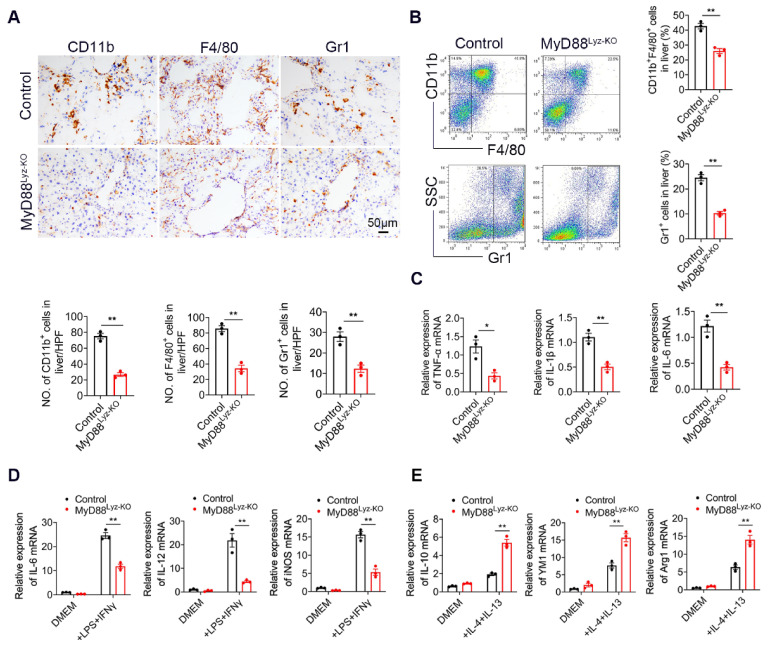
Myeloid cell-specific MyD88 deletion attenuates the liver inflammatory response. Groups of control and MyD88^Lyz-KO^ mice (*n* = 5 per group) were subjected to CCl_4_-induced liver fibrosis. The data are representative of at least three independent experiments. (**A**) Representative staining and statistical analysis of CD11b, F4/80, and Gr-1 in liver tissues. Scale bar, 50 μm. ** *p* < 0.01. (**B**) Isolation of liver lymphocytes from CCl_4_-induced control and MyD88^Lyz-KO^ mice and flow cytometry analysis of the proportion of CD11b^+^F4/80^+^ macrophages and Gr-1^+^ neutrophils in the livers after staining with F4/80, CD11b, and Gr-1 antibodies, ** *p* < 0.01. (**C**) The mRNA levels of TNF-α, IL-1β and IL-6 in liver tissues were measured using qPCR analysis. * *p* < 0.05 and ** *p* < 0.01. (**D**) Fold change of IL-6, IL-12 and iNOS mRNA in control and MyD88^Lyz-KO^ BMDMs polarized to M1 with LPS/IFN-γ for 24 h. ** *p* < 0.01. (**E**) Fold change of IL-10, YM1 and Arg1 mRNA in control and MyD88^Lyz-KO^ BMDMs polarized to M2 with IL-4/IL-13 for 48 h. ** *p* < 0.01.

To explore whether MyD88 signaling in myeloid cells regulates inflammatory cell infiltration during fibrosis, F4/80, CD11b and Gr1 immunohistochemical staining and flow cytometry were performed to evaluate inflammatory cell infiltration. As shown in Figure 3A,B, the infiltration of CD11b^+^ macrophages and F4/80^+^ macrophages were prominently decreased in liver tissues from MyD88^Lyz-KO^ mice compared with those from control mice following repetitive CCl_4_ injection. MyD88 deficiency in myeloid cells also reduced CCl_4_-induced Gr1^+^ neutrophil infiltration in livers compared with those in control mice. Furthermore, the qPCR analysis revealed that hepatic levels of the pro-inflammatory cytokines, such as TNF-α, IL-1β and IL-6, were also decreased significantly in MyD88^Lyz-KO^mice compared to those in control mice after CCl_4_ treatment (Figure 3C). 

To determine whether and how MyD88 participated in the regulation of macrophage polarization, we first determined the effect of MyD88 deficiency on BMDMs polarized to M1 and M2 macrophages with LPS/IFN-γ and IL-4/IL-13, respectively (Figure 3D,E). As shown in Figure 3D, MyD88 deletion markedly suppressed the expression of the M1-specific marker genes IL-6, IL-12 and iNOS upon LPS/IFN-γ stimulation. In contrast, MyD88 deletion significantly enhanced the M2-specific marker genes IL-10, YM1 and Arg1upon IL-4/IL-13 stimulation (Figure 3E). Collectively, these results demonstrated that MyD88 deletion in macrophages attenuates the CCl_4_-induced inflammatory response.

### 2.4. Specific Genetic Deletion of MyD88 in Macrophages Reduces CXCL2 Secretion

**Figure 4:**
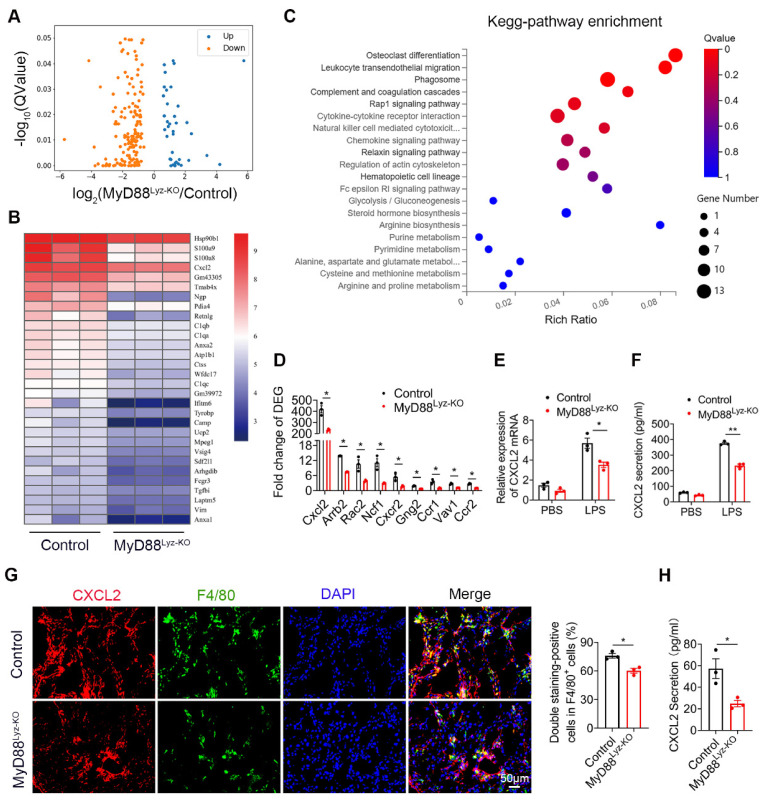
CXCL2 secreted by macrophages is critical for liver fibrosis. RNA sequencing analysis of DEGs between CCL_4_-induced liver tissues from control and MyD88^Lyz-KO^ mice. (**A**) Volcano diagram of DEGs, the threshold is padj < 0.05. (**B**) Heatmap view of the most significant differential expressed genes. (**C**) KEGG enriched signal pathway analysis with up-regulated genes. (**D**) Analysis of fold change of DEGs, * *p* < 0.05. (**E**) The relative RNA expression of CXCL2 in LPS treated BMDMs were measured by q-PCR, * *p* < 0.05. (**F**) The secretory protein levels of CXCL2 in LPS treated BMDMs were measured by ELISA, ** *p* < 0.01. (**G**) Double immunofluorescence staining and statistical analysis for F4/80 and CXCL2 in liver tissues. Scale bar, 50 μm. * *p* < 0.05. (**H**) ELISA verification for serum CXCL2 levels in MyD88*^Lyz-KO^* or control mice treated with CCL_4_. * *p* < 0.05.

To further compare detailed changes in the gene expression signature, we performed protein-coding mRNA-seq analysis of liver tissues from CCl_4_-treated MyD88^Lyz-KO^ mice and control mice. A total of 196 differentially expressed genes (DEGs) were identified, including 37 upregulated and 159 downregulated genes (Figure 4A; *p* < 0.05). Consistently, genes related to inflammation in control mice had higher expression compared with those in MyD88^Lyz-KO^ mice. The top 30 differential expressed genes were selected to be shown in a heat map (Figure 4B). Next, Pathway analysis showed that MyD88^Lyz-KO^ mice had significantly downregulated inflammation-related genes. In particular, the expression of CXCL2 was decreased significantly in CCl_4_-treated MyD88^Lyz-KO^mice compared with control mice (Figure 4C,D). qPCR analysis showed that the deletion of MyD88 partly suppressed LPS-induced CXCL2 expression in BMDMs (Figure 4E). The protein expression level of CXCL2 was further analyzed by ELISA of the BMDMs-conditioned media (CM). Deletion of MyD88 partly attenuated the expression of CXCL2 in LPS-induced BMDMs CM (Figure 4F). Consistent with all experimental results, the expression of CXCL2 in F4/80^+^cells was decreased significantly in MyD88^Lyz-KO^ mice in fibrotic liver tissues (Figure 4G). Furthermore, serum ELISA analysis revealed that CCL_4_-treated MyD88^Lyz-KO^mice also significantly reduced CXCL2 secretion compared with control mice (Figure 4H). These results indicate that macrophage-derived CXCL2 may play important roles in the process of liver fibrosis.

### 2.5. Macrophages Promote the Activation of HSCs by Secreting CXCL2

**Figure 5:**
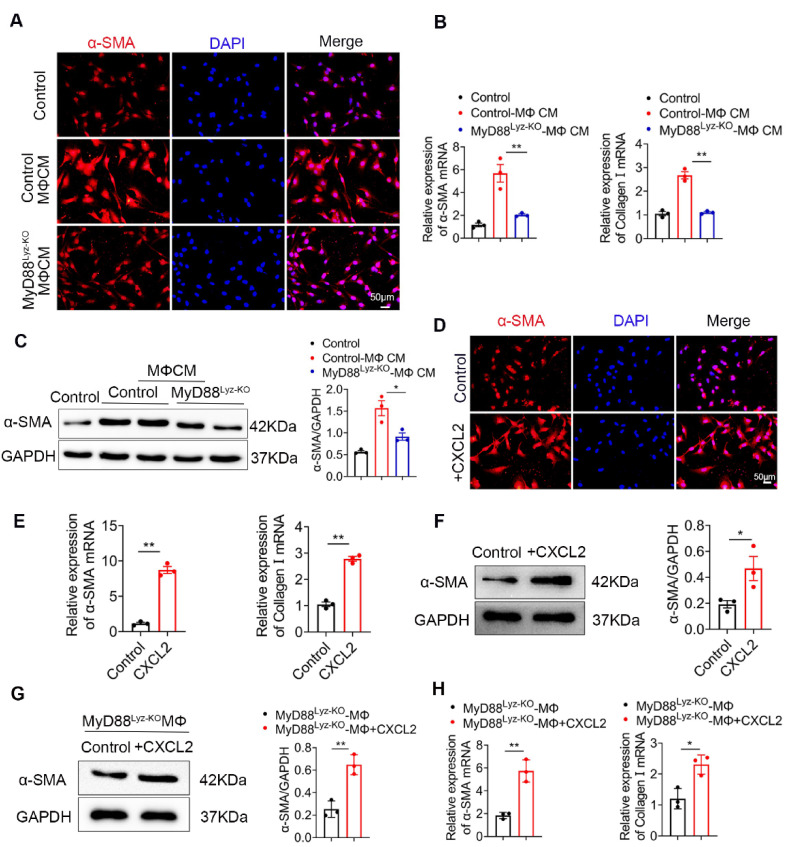
CXCL2 secreted by macrophages promotes the activation of HSCs. (**A**–**C**) LX-2 cells were cultured with control and MyD88^Lyz-KO^ mice BMDM CM for 24 h. (**A**) Immunofluorescence staining for α-SMA in LX-2 cells. Scale bar, 50 μm. (**B**) The mRNA levels of the activation-related genes α-SMA and collagen I in LX-2 cells were measured using qPCR analysis. ** *p* < 0.01. (**C**) The protein levels of α-SMA in LX-2 cells were detected by Western blot. The densities of proteins were quantified by densitometry. α-SMA were normalized to GAPDH. * *p* < 0.05. (**D**–**F**) LX-2 cells were treated with CXCL2 for 24 h. (**D**) Immunofluorescence staining for α-SMA in LX-2 cells. Scale bar, 50 μm. (**E**) The mRNA levels of α-SMA and collagen I in LX-2 cells were measured using qPCR analysis. ** *p* < 0.01. (**F**) The protein levels of α-SMA in LX-2 cells were detected by Western blot. The densities of proteins were quantified by densitometry. α-SMA were normalized to GAPDH. * *p* < 0.05. (**G**,**H**) LX-2 cells treated with exogenous CXCL2 co-cultured with BMDMs isolated from MyD88*^Lyz-KO^* mice for 24 h. (**G**) The protein levels of α-SMA in LX-2 cells were detected by Western blot. The densities of proteins were quantified by densitometry. α-SMA were normalized to GAPDH. ** *p* < 0.01. (**H**) The mRNA levels of α-SMA and collagen I in LX-2 cells were measured using qPCR analysis. * *p* < 0.05 and ** *p* < 0.01.

HSCs play important roles in liver fibrosis. We further investigated the effect of macrophage-derived CXCL2 on HSCs. To assess whether MyD88 in BMDMs could induce HSCs activation, human HSC cell line LX-2 cells were used and treated with the CM, which was collected from cultured BMDMs isolated from MyD88^Lyz-KO^ or control mice treated with CCL*_4_*. Following incubation for 24 h, α-SMA expression was analyzed by immunostaining. MyD88 deficiency in BMDMs significantly decreased the expression of α-SMA in LX-2 cells (Figure 5A). qPCR analysis showed that expression of α-SMA and collagen I was also significantly decreased in LX-2 cells treated with CM of MyD88 deficiency in BMDMs (Figure 5B). The Western blot also revealed similar results (Figure 5C).

Since the deletion of MyD88 in macrophages decreases the secretion of CXCL2, we wondered whether MyD88 in macrophages promotes the activation of HSCs through CXCL2. Next, LX-2 cells were cultured with exogenous addition of CXCL2 recombinant protein for 24 h. We found that the levels of α-SMA were increased significantly compared to those in the untreated group by immunostaining (Figure 5D). Consistently, qPCR and Western blot also revealed similar results (Figure 5E,F). Furthermore, Western blot and qPCR revealed that LX-2 cells treated with exogenous CXCL2 co-cultured with BMDMs isolated from MyD88*^Lyz-KO^* mice rescue α-SMA expression and HSCs activation (Figure 5G,H). These results indicate that MyD88 in macrophages promotes the activation of HSCs by secreting CXCL2.

### 2.6. CXCL2 Induced NLRP3 Inflammasome Activation in HSCs

**Figure 6:**
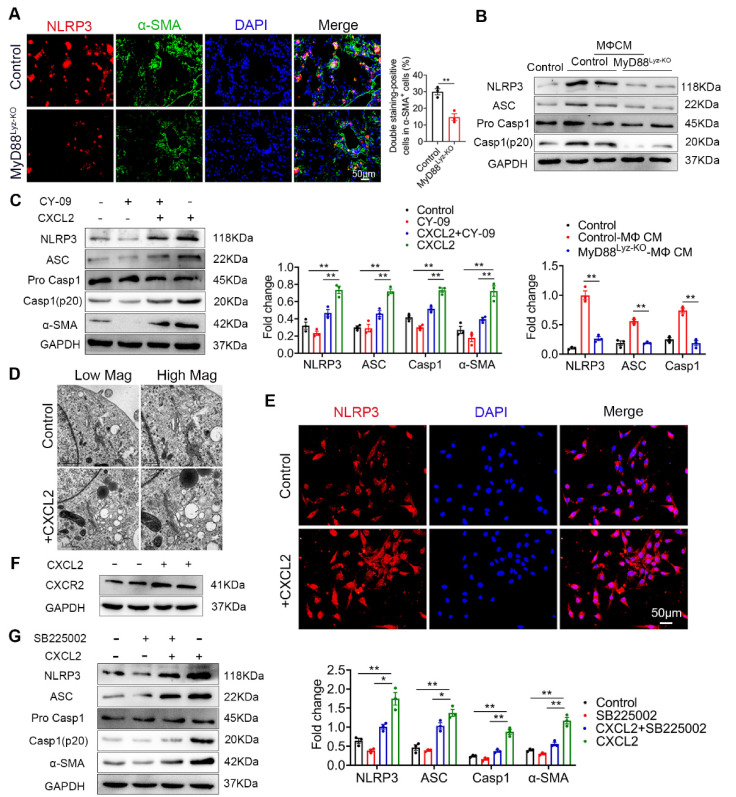
CXCL2 activates NLRP3 inflammasome in HSCs. (**A**) Groups of control and MyD88^Lyz-KO^ mice (*n* = 5 per group) were subjected to CCl_4_-induced liver fibrosis. Double immunofluorescence staining and statistical analysis for NLRP3 and α-SMA in liver tissues. Scale bar, 50 μm. ** *p* < 0.01. (**B**) Control and MyD88^Lyz-KO^ mice BMDM CM-treated LX-2 cells for 24 h; the expression levels of NLRP3 inflammasome-associated protein were detected by Western blot analysis. The densities of proteins were quantified by densitometry. NLRP3, Cleaved-Caspase 1 and ASC were normalized to GAPDH. ** *p* < 0.01. (**C**) After pretreatment of LX-2 cells with or without CY-09 (NLRP3 inhibitor) for 2 h, 200 ng/mL CXCL2 recombinant protein was incubated for 24 h, and the expression of NLRP3 inflammasome-associated protein was detected by Western blot analysis. The densities of proteins were quantified by densitometry. NLRP3, Cleaved-Caspase 1 and ASC were normalized to GAPDH. ** *p* < 0.01. (**D**) Representative electron microscopy of LX-2 cells treated with or without 300 ng/mL CXCL2 for 24 h. Scale bar, 1 μm (left). Scale bar, 0.5 μm (right). (**E**) Immunofluorescence staining for NLRP3 in LX-2 cells. Scale bar, 50 μm. (**F**) cells were stimulated with 200 ng/mL CXCL2 recombinant protein for 24 h, and CXCR2 protein level was determined by Western blot analysis. (**G**) LX-2 cells were pretreated with or without SB225002 (CXCR2 inhibitor) for 30 min, and then treated with CXCL2 for 24 h, and the expression levels of NLRP3 inflammasome-related proteins were detected by Western blot analysis. The densities of proteins were quantified by densitometry. NLRP3, Cleaved-Caspase 1 and ASC were normalized to GAPDH. The data are representative of at least three independent experiments. * *p* < 0.05 and ** *p* < 0.01.

Inflammasome activation had been associated with several chronic liver diseases, including fibrosis development [25]. Some studies reported that NLRP3 activation was required for hepatic inflammation and fibrosis [26,27]. We wonder whether the NLRP3 inflammasome activated in HSCs directly contributed to liver fibrosis development. As shown in Figure 6A, the activation of NLRP3 in α-SMA^+^ cells was significantly weakened in MyD88^Lyz-KO^ mice in fibrotic liver tissues. Inhibition of inflammasome activation may be an important cause of attenuated liver fibrosis in mice with myeloid-specific deletion of MyD88. Then, the HSC cell line LX-2 cells were stimulated with BMDM CM of control and MyD88^Lyz-KO^ mice and NLRP3 inflammasome related proteins were analyzed by Western blot. As a result, MyD88 deficiency in BMDMs significantly decreased the expression of NLRP3, Cleaved-Caspase 1 and ASC in LX-2 cells (Figure 6B). To further identify the molecular mechanisms underlying the effects of extracellular CXCL2 on the activation of the inflammasome, LX-2 cells were cultured with CXCL2 recombinant protein and NLRP3 inflammasome related proteins were analyzed by Western blot (Figure 6C). We found that NLRP3, Cleaved-Caspase 1 and ASC were significantly upregulated by CXCL2 protein. When NLRP3 activation was inhibited with NLRP3 inhibitor CY-09 in LX-2 cells, CXCL2-induced NLRP3, Cleaved-Caspase 1, ASC and α-SMA expression were weakened (Figure 6C). The results demonstrated that CXCL2 promotes HSC activation by activating NLRP3 inflammasome. Previous studies reported that NLRP3 puncta were formed on the outside of vesicle-like structures [28]. Furthermore, we also observed that in LX-2 cells stimulated with recombinant protein CXCL2, a number of giant vesicles appeared in the perinuclear (Figure 6D). Moreover, immunofluorescence staining showed that CXCL2 stimulation significantly enhanced the activation of NLRP3 in LX-2 cells (Figure 6E). 

CXCL2 functions via its specific receptor CXCR2. Next, we test whether CXCL2-induced NLRP3 inflammasome in HSCs depends on CXCR2. As shown in Figure 6F, we observed the expression of CXCR2 on HSCs. Furthermore, we used a CXCR2-specific inhibitor (SB225002) to prevent CXCR2 activation in CXCL2-cultured HSCs. As a result, the CXCR2 inhibitor significantly inhibited NLRP3-mediated activation of HSCs (Figure 6G). These results showed that CXCL2 induces HSCs activation via NLRP3 inflammasome.

### 2.7. Targeting CXCL2 with a CXCR2 Inhibitor Effectively Attenuates CCl_4_-Induced Liver Fibrosis

**Figure 7:**
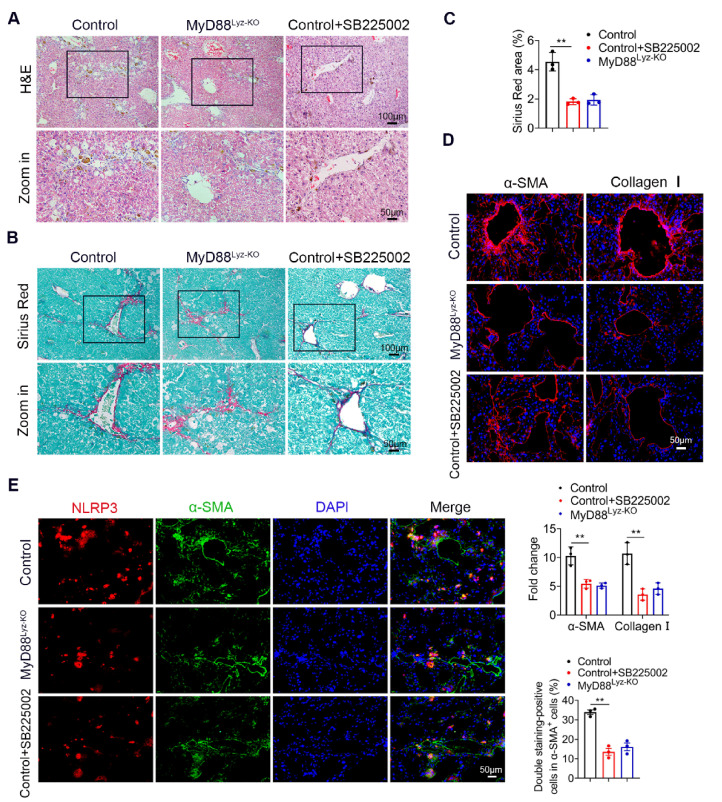
CXCR2 inhibitor effectively attenuates CCl_4_-induced liver fibrosis. Groups of control mice (*n* = 5 per group) were treated with CCL_4_ and were injected intraperitoneally with SB225002 (2 mg/kg) twice a week or oil as control for 4 weeks. MyD88^Lyz-KO^ mice were injected with CCl_4_ at the same time. The data are representative of at least three independent experiments. (**A**) Representative staining of H&E in liver tissues. Scale bar, 100 μm (top). Scale bar, 50 μm (bottom). (**B**) Liver tissues were harvested and stained with Sirius Red. Scale bar, 100 μm (top). Scale bar, 50 μm (bottom). (**C**) Statistical analysis of Sirius Red staining in liver tissues. ** *p* < 0.01. (**D**) Representative staining and statistical analysis of α-SMA and collagen I in liver tissues. Scale bar, 50 μm. ** *p* < 0.01. (**E**) Double immunofluorescence staining and statistical analysis for NLRP3 and α-SMA in liver tissues. Scale bar, 50 μm. ** *p* < 0.01.

To further investigate the role of CXCL2 in liver fibrosis, we examined the therapeutic potential of a CXCR2 inhibitor in a mouse liver fibrosis model. By administering CXCR2 inhibitor (SB225002) or Oil preventively to CCl_4_-treated mice, we found that SB225002 significantly weakened liver injury and attenuated CCl_4_-induced liver fibrosis by H&E staining (Figure 7A) and Sirius Red staining (Figure 7B,C). Consistently, less hepatic expression of α-SMA and collagen I were found after the inhibitor of CXCR2 treatment (Figure 7D). More importantly, CXCR2 inhibitor significantly inhibited the activation of NLRP3 in α-SMA^+^ cells revealed by double immunofluorescence staining (Figure 7E). These results indicated that targeting CXCL2 by CXCR2 inhibitors could be an efficient treatment for liver fibrosis. CXCL2 may be a promising target for the prevention and treatment of liver fibrosis.

## 3. Discussion

Hepatic fibrogenesis involves multiple recruited cell populations resulting in an aberrant wound-healing response characterized by the accumulation of the ECM. Macrophages play an important role in the wound healing response, including the promotion of HSCs activation. In this study, the role of MyD88 signaling in macrophages in the pathogenesis of liver fibrosis was investigated. Deletion of MyD88 in myeloid cells attenuated liver fibrosis by reducing inflammatory cell infiltration. In vitro, inhibition of MyD88 signaling in macrophages reduced the secretion of CXCL2, which in turn inhibited the activation of HSCs. Furthermore, mechanistic studies found that CXCL2 induced NLRP3 inflammasome activation in HSCs. Thus, MyD88 in macrophages may represent a potential candidate for the prevention and therapy of liver fibrosis.

A series of studies on the relationship between hepatic inflammation and fibrogenesis have been reported. TLRs are pattern recognition receptors that perceive pathogens and bacteria-derived molecules, leading to proinflammatory cytokine production [29]. MyD88 is a key downstream adapter for most TLRs. TLR4 can activate fibroblasts, promote fibrogenesis in numerous organs, including the liver, lungs, kidneys, heart, and skin via MyD88-dependent and TRIF-dependent pathways [30,31,32,33]. Especially, TLR4-MyD88 pathway promoted liver fibrosis in a mouse BDL model by enhancing TGF-β signaling [20]. In this study, we first reported the myeloid cell-specific role of MyD88 signaling in liver fibrosis. Our results demonstrated that MyD88 in myeloid cellsenhanced hepatic fibrosis.

Macrophages hold a central position in the pathogenesis of chronic liver injury and have been proposed as potential targets in combatting fibrosis [17]. On the one hand, macrophages are major regulators of inflammation and fibrogenesis by regulating the crosstalk between HSCs and immune system cells to achieve a cellular response [34,35]. There is clear evidence from in vitro and in vivo studies that Kupffer cells can activate HSCs to transdifferentiate into myofibroblasts, the major collagen-producing cell type in hepatic fibrosis [36]. On the other hand, macrophages produce cytokines and chemokines that directly activate fibroblasts and recruit inflammatory cells [37]. Hepatic macrophages interact with other immune cells; for instance, they secrete the chemokine CXCL16 that attracts NKT cells, which in turn can activate pro-inflammatory signals in macrophages [38]. CXCL2, also known as macrophage inflammation protein 2 (MIP-2), is a small, secreted cytokine that belongs to the CXC chemokine family [39,40]. CXCL2 is mainly expressed on macrophages, endothelial cells, and glial cells. The principal role of CXCL2 involves the chemotaxis of neutrophils into inflamed tissues, where they bind to the chemokine receptor CXCR2 [41,42]. However, studies that focus on the relationship between CXCL2 and liver fibrosis are rare. Here, our results indicated that MyD88 in macrophages activates HSCs through CXCL2 secretion and promotes liver fibrosis.

Liver fibrosis is characterized by abnormal collagen accumulation and activation of HSCs. The activation of HSCs is always considered as the central event during the progression of liver fibrosis [1,43]. Therefore, HSCs are commonly regarded as the targets for antifibrotic therapies recent years [44]. It has been reported that NLRP3 inflammasome plays an important role in liver fibrosis development. However, the mechanisms involved in NLRP3-induced fibrosis are still unclear. Growing evidence supports a central role of NLRP3 activation and downstream effectors in the development of liver fibrosis [25,26,27,45,46]. It has been shown that the level of NLRP3 expression, among other inflammasomes, is augmented during experimental liver fibrosis [47]. To date, there are only a few studies elucidating different stimuli that can trigger HSC activation and subsequent upregulation of fibrotic markers along with the activation of the NLRP3 inflammasome in both LX-2 cells, an immortalized human stellate cell line, and murine primary HSC [48,49,50]. Our results indicated that the NLRP3 inflammasome in HSCs can directly regulate their activation and contribute to liver fibrosis. Consistent with these results, our results further confirmed that NLRP3 inflammasome activation in HSCs and promoted the progression of liver fibrosis.

**Figure 8:**
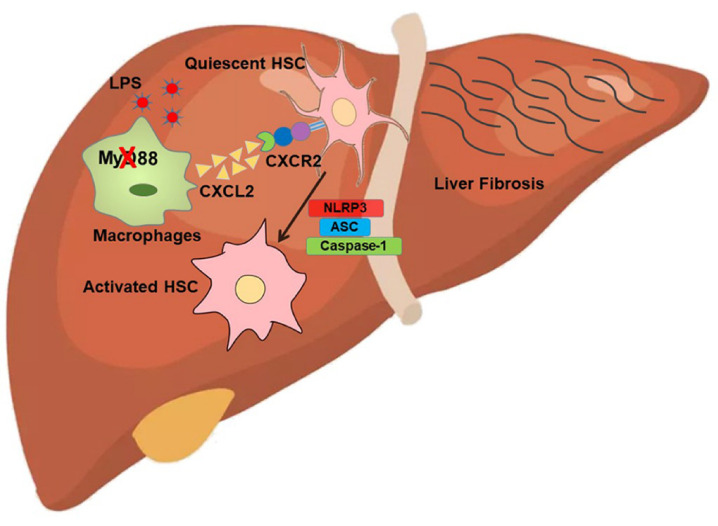
Schematic of MyD88 signaling in macrophages in CCL_4_-induced liver fibrosis model. Deficiency of MyD88 in macrophages reduces the secretion of CXCL2, thus inhibiting the activation of HSC and fibrosis, which is characterized by the activation of NLRP3 inflammasomes.

In conclusion, our study demonstrated that MyD88 in macrophages promoted the development of liver fibrosis via activating NLRP3 inflammasome in HSCs. Inhibition of the key factors on the axis MyD88/TLR-CXCL2-CXCR2-NLRP3 may provide potential prevention and treatment strategies for liver fibrosis (Figure 8).

## 4. Materials and Methods

### 4.1. Mice

MyD88^flox/flox^ and lyz-Cre mice on a C57BL/6 background were purchased from Jackson Laboratory (Bar Harbor, ME, USA). Mice with a conditional knockout of MyD88 in lyz-expressing myeloid cells (MyD88^Lyz-KO^ mice) were generated by crossing MyD88^flox/flox^ and lyz-Cre mice. All mice were maintained in specific pathogen-free and humidity- and temperature-controlled microisolator cages with a 12 h light/dark cycle at the Institute of Biophysics, Chinese Academy of Sciences. MyD88^Lyz-KO^ mice and their littermate controls used for the experiments were 6 to 8 weeks old. All animal studies were performed after being approved by the Institutional Laboratory Animal Care and Use Committee of the Institute of Biophysics, Chinese Academy of Sciences.

### 4.2. CCl_4_-Induced Liver Fibrosis

CCl_4_ was mixed with common corn oil (Sigma-Aldrich, St. Louis, MO, USA) at a 1:9 ratio and injected intraperitoneally (i.p.) into mice at 0.5 μL CCl_4_/g body weight twice per week for four weeks as described previously [51]. Five animals per group or condition were used. Experiments were performed at least three times.

The CXCR2 antagonist SB225002 (MedChemExpress, Princeton, NJ, USA) or control corn oil was injected i.p. at 2 mg/kg body weight twice per week. The injection started 24 h before CCl_4_ administration and lasted for 4 weeks.

### 4.3. Cell Lines and Treatments

The LX-2 cell line was purchased from Xiangya Medical College (Changsha, China). The cells were cultured in DMEM (HyClone, Logan, UT, USA) supplemented with 10% fetal bovine serum (FBS, HyClone) and 1% penicillin/streptomycin. Cells were cultured at 37 °C with 5% CO_2_. LX-2 cells were exposed to inhibitor of NLRP3 CY-09 (10 μM) (MedChemExpress, Princeton, NJ, USA) for 2 h. After incubation, the cells were challenged with 200 ng/mL CXCL2 recombinant protein (Sino Biological, Beijing, China) for 24 h for further analysis. LX-2 cells were treated with 200 ng/mL CXCL2 recombinant protein and 400 nM inhibitor of CXCL2 receptor CXCR2 SB225002 for 24 h for further analysis.

### 4.4. Blood Biochemical Assays

Mouse blood samples were centrifuged at 3000 g for 8 min to obtain serum. Serum ALT and AST levels were detected at the Vitonglihua Experimental Animal Centre (Beijing, China).

### 4.5. Histology and Immunostaining

Preparation of paraffin or cryostat tissue sections was performed as described previously [52]. The sliced liver paraffin sections were then stained with H&E and Sirius Red. For immunohistochemistry (IHC), cryostat sections were incubated with anti-F4/80, anti-CD11b and anti-Gr-1 antibodies (BD Pharmingen, San Diego, CA, USA) followed by incubation with horseradish peroxidase (HRP)-conjugated secondary antibodies. For fluorescence staining, cryostat sections were incubated with anti-MyD88, anti-α-SMA, anti-collagen I, anti-NLRP3, and anti-CXCL2 antibodies (ProteinTech, Chicago, IL, USA) followed by incubation with Alexa Fluor 488-conjugated secondary antibodies (Invitrogen, Carlsbad, CA, USA). Sections were evaluated under a microscope (DP71, OLYMPUS, Tokyo, Japan) for bright-field and fluorescence microscopy.

### 4.6. Isolation of Monocyte-Derived Macrophages

Bone marrow cells were isolated from the femur and tibia of 8-week-old male C57BL/6 mice. To obtain macrophage colony-stimulating factor (mCSF) from fibroblast conditioned medium (FCM), L929 fibroblasts were cultured in RPMI 1640 medium with 10% FBS for 3 d and the supernatant FCM was collected and centrifuged at 12,000× *g* for 10 min. The centrifuged FCM was stored at −80 °C. Bone marrow cells were cultured in a DMEM medium containing 10% FBS and 20% FCM for 1 week to generate BMMs. By the 7th day, all adherent cells had become mature macrophages [53,54].

### 4.7. Collection of Conditioned Media

To obtain conditioned media, cells were grown to subconfluence in 10 cm culture plates. The culture media was changed to serum-free media. After 48 h, the media were harvested, and debris was removed by centrifugation at 1500 rpm for 3 min at 4 °C.

### 4.8. Cellular Immunofluorescence

The LX-2 cells were plated into 96-well plates (1 × 10^3^ cells/well). For fluorescence staining, the cells were fixed in 4% paraformaldehyde for 15 min and permeabilized with 0.2% Triton-X 100 for 10 min at room temperature. The cells were incubated with 2% BSA to block nonspecific binding sites. Then, the cells were incubated with the anti-α-SMA, and anti-NLRP3 antibodies (Abcam, Cambridge, UK) followed by incubation with Alexa Fluor 488- or 594-conjugated secondary antibodies (Invitrogen, Carlsbad, CA, USA). 

### 4.9. Flow Cytometry Analysis

Single-cell suspensions were prepared from liver NPCs and stained with the following directly labeled mouse-specific mAbs: Percp/Cy5.5-labeled anti-CD11b (clone M1/70), APC-labeled anti-F4/80 (clone BM8) and FITC-labeled anti-Gr-1 (clone RB6-8C5). Antibodies were purchased from Biolegend and used at a 0.2 μg/mL concentration. Cells were collected on a FACS Calibur (BD Biosciences, San Diego, CA, USA) and analyzed by FlowJo software (TreeStar, Ashland, OR, USA).

### 4.10. Transmission Electron Microscopy

The LX-2 cells were cultured with or without 300 ng/mL CXCL2 recombinant protein for 24 h. Then cells were fixed in 2% glutaraldehyde and 2.5% glutaraldehyde post-fixed in 1% osmium tetroxide, rinsed with sodium cacodylate trihydrate buffer, dehydrated with gradient alcohol, which was then replaced with propylene oxide and embedded in Epon 812. Semi-thin (1 µm) sections were cut, stained with methylene blue and then examined under a microscope. Ultra-thin sections were stained with lead citrate and uranyl acetate and were examined using a JEM-1400 electron microscope (JEOL, Tokyo, Japan). TEM images of the sections were taken using a camera-specific imaging system for EM (830.10U3 CCD Gatan, Pleasanton, CA, USA).

### 4.11. Quantitative Real-Time Polymerase Chain Reaction (qPCR)

Total RNA was isolated from frozen liver pieces or cells using TRIzol reagent (TransGen Biotech, Beijing, China). cDNA was synthesized using a Primescript RT Master Mix Kit (MedChemExpress, Princeton, NJ, USA). qPCR was performed in duplicate with a SYBR Premix Ex TaqTM Kit (MedChemExpress, Princeton, NJ, USA). Data were analyzed using the 2-ΔΔCt method and normalized to GAPDH expression [55].

### 4.12. Western Blot Analysis

Western blotting was performed as previously described [56]. Cultured cells and dissected tissues were collected, and protein was extracted using RIPA lysis buffer (Beyotime, Shanghai, China) containing a cocktail protease inhibitor (Biotool, Houston, TX, USA). Samples were incubated at 99 °C for 5 min and separated by electrophoresis on a 10% SDS-PAGE gel at 115 V for 1.2 h. Proteins were transferred to a PVDF membrane at 200 mA for 1 h. Membranes were blocked with 5% milk in TBST at RT for 1 h and incubated overnight at 4 °C with the following primary antibodies: anti-NLRP3, anti-ASC, anti-caspase1, anti-CXCR2 (ProteinTech, Chicago, IL, USA), anti-GAPDH, anti-α-SMA (Santa Cruz Biotechnology, Santa Cruz, CA, USA). HRP-conjugated goat anti-mouse IgG and goat anti-rabbit IgG were used as secondary antibodies. Blots were scanned using a Clinx Science Instrument. All specific bands were quantified with an Automated Digitizing System (ImageJ 1.8.0).

### 4.13. ELISA

Primary macrophages were incubated with LPS (10 μg/mL) for 24 h. After activation, the cells were rinsed with PBS and cultured in fresh serum-free medium. After 24 h, the supernatant was harvested and used for subsequent ELISA determination. The CXCL2 ELISA kit was purchased from BIOSs (Shanghai, China). All tests were carried out according to the manufacturer’s instructions.

### 4.14. RNA Sequencing Analysis 

RNA-sequencing analyses were performed in fibrotic liver tissues from control and MyD88^Lyz-KO^ mice. Total RNA was extracted with RNeasy Mini Kit (QIAGEN, Dusseldorf, Germany), and RNA-sequencing analyses were performed on the BGISEQ-500 sequencer platform by BGI (Shenzhen, China). Stats package and plots with ggplot2 package in R (version 3.5) were used in principle component analysis. The raw transcriptomic reads were mapped to Nipponbare reference genome using HISAT40/Bowtie241 tools after removing adaptor sequences, reads containing polyN sequences, and low-quality reads. Normalization was performed and RESM software was used. Significantly differentially expressed genes (DEGs) were identified by setting padj <0.05, and the absolute value of log2 Ratio ≤ 0.5. The KEGG (Kyoto Encyclopedia of Genes and Genomes) enrichment analysis was performed by using phyper in R. All data mining, and figure presentation were conducted on the Dr Tom network platform of BGI (http://report.bgi.com).

### 4.15. Statistical Analysis

All data were expressed as the mean ± SEM and analyzed using GraphPad Prism software 8.0.2. Significant differences between mean values were obtained using three independent experiments. Differences between the two groups were compared using two-tailed unpaired Student’s *t*-test analysis. One-way ANOVA with Bonferroni correction was used for multiple comparisons. *p* < 0.05 was considered statistically significant.

## Data Availability

The datasets utilized in the present study are available from the corresponding author on reasonable request.
